# The Itappachi Universal Motion Platform for Accurate Dose Measurement in Thoracoabdominal Radiotherapy

**DOI:** 10.7759/cureus.71713

**Published:** 2024-10-17

**Authors:** Naoki Tohyama, Eriko Saito, Kazuhide Uchida, Kiyoshi Yoda, Shinichiro Mori

**Affiliations:** 1 Department of Radiological Sciences, Komazawa University, Tokyo, JPN; 2 Department of Radiation Oncology, Tokyo Bay Makuhari Clinic for Advanced Imaging, Cancer Screening, and High-Precision Radiotherapy, Chiba, JPN; 3 Corporate Research and Development Center, Perfect Imaging Laboratory, Chiba, JPN; 4 Corporate Research and Development Center, Anzai Medical Co. Ltd., Tokyo, JPN; 5 Research Center, Perfect Imaging Laboratory, Chiba, JPN

**Keywords:** dose measurement, photon beam, radiation therapy, respiratory gating, vmat

## Abstract

We developed the “Itappachi” universal motion platform for measuring radiation doses under simulated respiratory motion in radiation therapy. The interplay effect, resulting from respiratory motion, degrades dose delivery precision in advanced treatments such as volumetric modulated arc therapy. The Itappachi platform is designed for precise dose measurement in dynamic scenarios through its ability to simulate respiratory motion. The platform features a large surface (580 mm × 380 mm), capable of supporting weights up to 56.8 kg, and moves with an amplitude of ±25 mm. It requires no silicone oil for maintenance, and it is controlled via Wi-Fi using user-owned devices, thereby reducing costs. Its motion accuracy was confirmed, with a maximum displacement error of 0.12 mm.

Using the platform, we conducted dose measurements with a Delta4 Phantom under static, moving without gated irradiation, and moving with gated irradiation conditions. Gamma index analysis revealed excellent agreement for static and dynamic gated conditions (99.4%), while significant dose degradation occurred in the non-gated dynamic condition (32.6%). The Itappachi platform provides a cost-effective and accurate solution for dose measurement under respiratory motion by which it supports improved quality assurance in radiation therapy.

## Introduction

Recent progress for technological developments in photon beam treatment such as volumetric modulated arc therapy (VMAT) are highly optimized therapies that are expected to have fewer side effects and better outcomes than conventional treatments due to their enhanced precision [1]. VMAT modifies the dose fluence map at each gantry angle. If the tumor position changes due to respiratory motion, the actual dose delivered during treatment may differ from the planned dose. This discrepancy can result in dose degradation, a phenomenon known as the interplay effect [2-4]. Therefore, it is necessary to further measure the difference between the calculated dose in the treatment plan and the actual delivered dose before starting the treatment.

A dosimeter is often put on a motion platform for precise dose measurement under actual treatment conditions. Due to time efficiency, two-dimensional (2D) and three-dimensional (3D) dosimeters, such as the Delta4 Phantom+, are commonly used for quality assurance dose measurements in place of measuring dose distribution at multiple points using a pinpoint chamber. A motion platform with a minimum length of 40 cm is needed for the Delta4 Phantom, which is approximately 40 cm long.

Currently, there are a few commercially available motion platforms, including the Dynamic Platform (Sun Nuclear Corporation, Melbourne, FL, USA), Enhanced Dynamic Platform (Sun Nuclear Corporation, Melbourne, FL, USA), QUASAR (Modus Medical Devices, Inc., London, ON, Canada), and PHANTOM 4X (ISP System, Vic en Bigorre, France). However, due to limitations in the specifications and the cost of motion platforms, only a small number of products can support the Delta4 Phantom+. A six-dimensional motion platform (HexaMotion, ScandiDos, Uppsala, Sweden) for moving the Delta4 Phantom+ is commercially available; however, as the platform is specifically designed for the Delta4 Phantom+, its use is not cost-effective.

To address this issue, we have developed a new motion platform, the “Itappachi,” and evaluated its basic performance in dose measurement during respiratory motion.

## Technical report

The Itappachi motion platform

**Figure 1:**
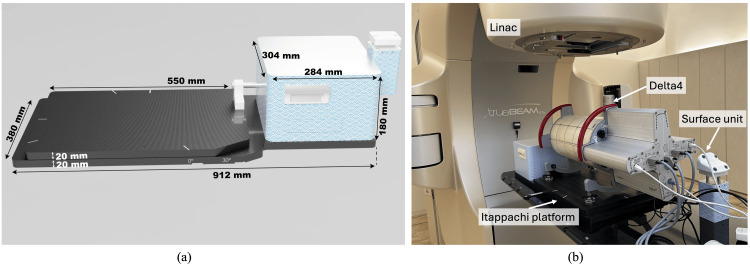
Dose measurement with the Delta4 Phantom on the Itappachi respiratory motion platform. (a) Photo of the Itappachi platform used to simulate respiratory motion, including its dimensions. (b) Experimental setup for dose measurement using the Delta4 Phantom+ device placed on the Itappachi platform. The setup illustrates the configuration used for both static and dynamic moving conditions, with and without gating, to evaluate the impact of respiratory motion on dose distribution accuracy. High energy X-ray irradiated by Linac (TrueBeam STx system).

The Itappachi motion platform (Perfect Imaging Laboratory, Chiba, Japan) consists of two plates and a control unit (Figure 1, Panel a). The upper plate moves in a single direction and can simulate respiratory motion with a waveform, achieving a cycle time of at least one second and an amplitude of ±25 mm. The upper plate, with its large size of 580 mm (length) × 380 mm (width), can accommodate various sizes of dosimeters and phantoms (Table 1). Rails are embedded in both the upper and lower plates, minimizing friction between the two, and allowing the upper plate to support objects weighing up to 52 kg. The upper plate can be moved using the following waveform patterns: Sin, Cos4, Cos6, patient-specific waveforms, including those from the Real-time Position Management (RPM) system (Varian Medical Systems Inc., Palo Alto, CA, USA), AZ (Anzai Medical Co., Ltd., Tokyo, Japan), and custom waveforms. The user can control the Itappachi motion platform via Wi-Fi using their own laptops or tablets without the need to install specific software, thereby ensuring ease of operation. Furthermore, as there is no need to purchase a separate laptop, this leads to cost savings.

**Table 1:** Comparison of specifications for the Itappachi motion platform and commercial alternatives. Specifications for the Itappachi motion platform and other commercially available products.

Product name	Itappachi platform	Dynamic platform [5]	Enhanced Dynamic platform [6]	QUASAR Heavy Duty Respiratory motion platform [7]	PHANTOM 4X 4X [8]
Vendor	Perfect Imaging Laboratory	Sun Nuclear	Sun Nuclear	Modus Medical Devices	ISP System
Plate dimensions	38 × 55 cm	35 × 35 cm	37.5 × 60 cm	35 × 45 cm	40 × 55 cm
Maximum motion	±25 mm	±25 mm	±25 mm	±20 mm	±41 mm
Maximum plate load	56.8 kg	32 kg	22.7 kg	45 kg	35 kg
Motion accuracy	±0.1 mm	±0.1 mm	±0.25 mm	Sub-millimeter	±0.1 mm
Overall weight	11 kg	17.2 kg	18.4 kg	8 kg	60 kg
Connection to controller	User-owned PC/tablet via Wi-Fi	Vendor-provided PC via Ethernet cable			
Control software	No specific software required	Vendor-provided software installation required			

The accuracy of the upper plate displacement may influence the overall results. To assess this, we shifted the phantom position by ±10 mm in 2.0 mm increments and measured the displacement using a calibrated laser sensor (HL-G2, Panasonic Industrial Devices, Kasugai, Japan), which has a nominal accuracy of 15 μm. A 56.8 kg weight was placed on the upper plate, and the measurements were repeated three times. The maximum displacement error recorded for the Itappachi platform was 0.12 mm, while the average positional error was 0.07 ± 0.04 mm.

Dose measurement

Treatment Planning

We acquired computed tomography (CT) scans with a slice thickness of 2.5 mm for treatment planning of the 28.5 cm × 23.0 cm × 20.0 cm water phantom using the Optima CT 580W CT system (GE Healthcare, Waukesha, WI). A 25.0 cm × 10.0 cm × 15.0 cm cork and a 3 cm diameter spherical mock tumor were placed at the center of the water phantom. One medical physicist contoured body, lung, clinical target volume (CTV), and planning target volume (PTV) in the treatment planning system (Eclipse Treatment Planning System Ver.16.1, Varian Medical Systems). CTV was defined as the 3 cm diameter mock tumor. The PTV was drawn by adding a margin of 0.5 cm in all directions to the CTV. The goal of the treatment plans was to cover the PTV with 95% of the prescribed dose of 12 Gy. VMAT was designed using a 6 MV flattening filter-free (FFF) beam with two partial arcs.

We measured dose distribution using the Delta4 Phantom, which was placed on the Itappachi platform, and compared the results across the following three conditions: static, moving without gated irradiation, and moving with gated irradiation. The Itappachi platform and surface unit were positioned on the treatment couch and moved sinusoidally (following a cos⁴ function) by ±10 mm with a four-second cycle (Figure 1, Panel b). The motion of the surface unit was tracked using the Varian RPM system, which was monitored by an infrared camera. Irradiation was performed using a TrueBeam STx system (Varian Medical Systems) with a 6 MV FFF X-ray beam. The gating window was set from 30% to 70%, corresponding to the flat region of the cos⁴ function.

Evaluation

We evaluated the gamma index method under the 3 mm/2% criterion, which measures the spatial and dose differences between a reference point and the evaluated point. These metrics can be calculated as follows:

$\Gamma = \min \sqrt{\frac{(D_r - D_e)^2}{\delta D^2} + \frac{(d_r - d)^2}{\delta d^2}}$

where dr and de are the position of the reference and evaluated doses Dr and De, respectively, and δD (%) and δd (mm) are the percentage dose difference and distance-to-agreement, respectively. δD and δd are often reported as δD/δd. This metric reduces sensitivity to errors, enhancing its robustness in managing discrepancies [9]. The pass rate is the percentage of points where the gamma index is less than 1. The average and maximum gamma index represent the mean and highest values of the gamma index, respectively.

**Figure 2:**
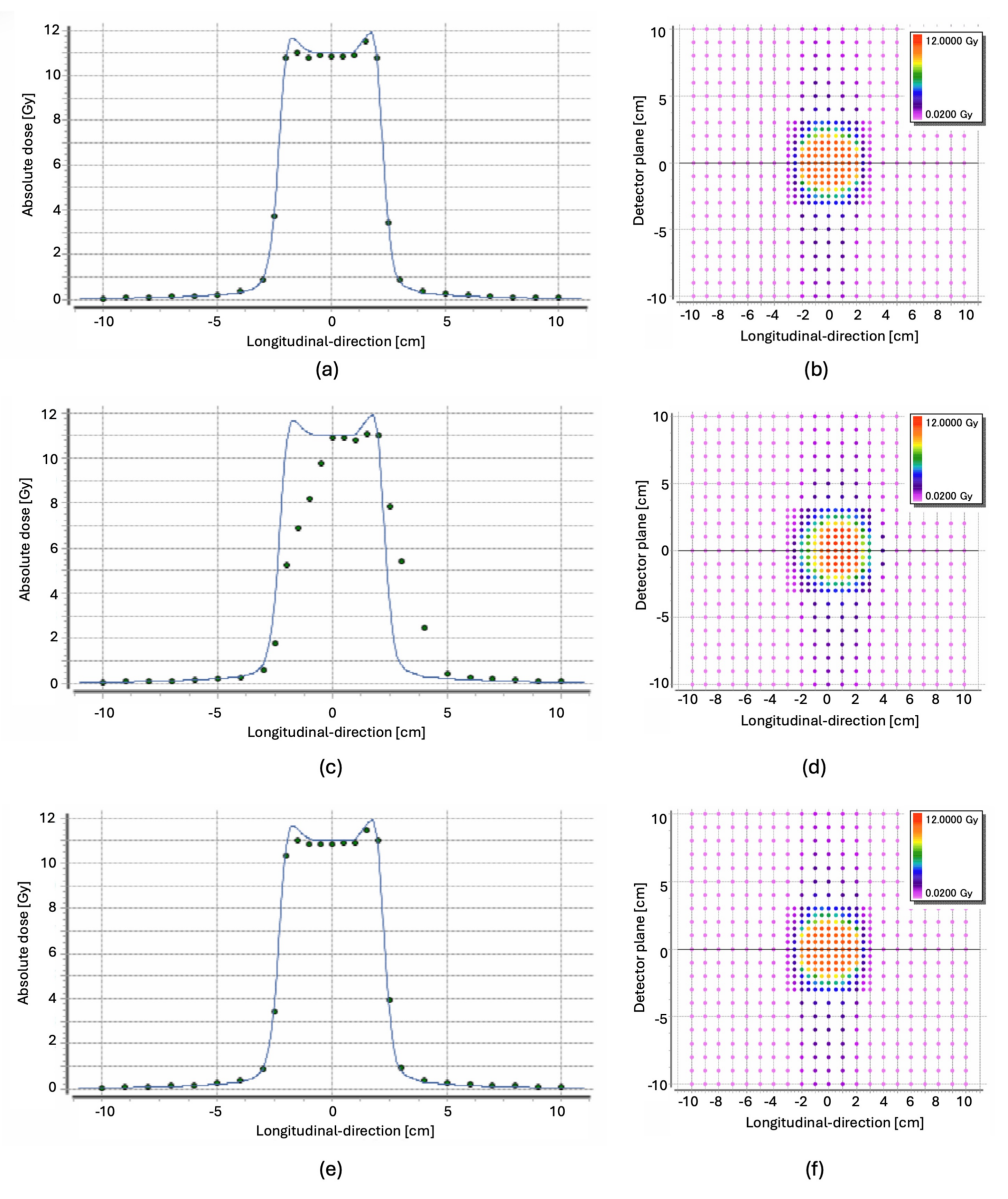
Comparison of dose profiles and two-dimensional distributions under static and motion conditions with and without gating. Dose profiles along the longitudinal direction (detector plane positioned at 0 cm) for the following three conditions: (a) static irradiation, (b) moving without gating, and (c) moving with gating. Solid lines and points were calculated and measured values, respectively. Color map visualizations are presented to compare the two-dimensional dose distributions at the respective measurement points for each condition: (d) static, (e) moving without gating, and (f) moving with gating.

**Figure 3:**
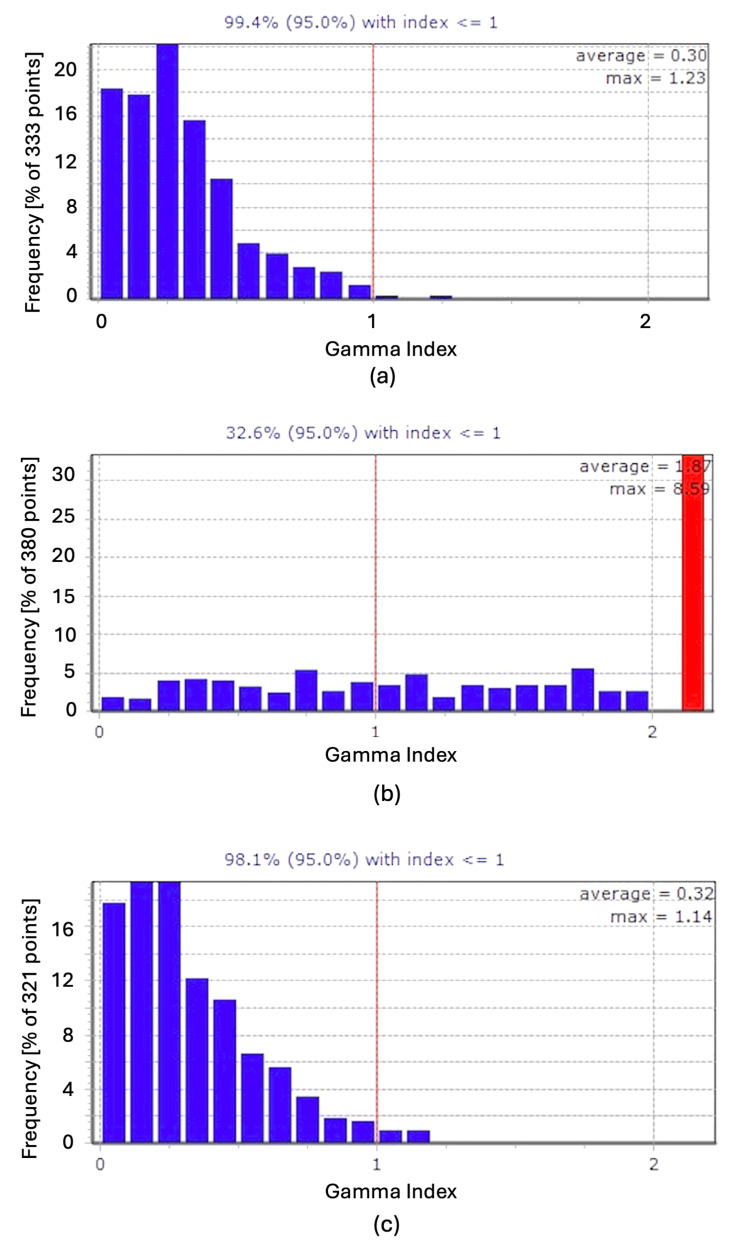
Gamma index histograms for static and motion conditions with and without gating. Histograms of the gamma index values for the three conditions evaluated: (a) static irradiation, (b) moving without gating (red bar shows more than 2 of the gamma index), and (c) moving with gating. The vertical red line shows the reference value (=1) of the gamma index. Frequency was calculated as more than 10% dose of the prescribed dose.

In the static condition, the measured and calculated dose profiles showed good agreement (Figure 2, Panel a), with a gamma index of 99.4% (Figure 3, Panel a). The maximum gamma index value was 1.23, and the average was 0.3, indicating a high level of dose agreement.

Regarding the dynamic moving condition without gating, the measured dose varied along the longitudinal direction compared to the planned dose due to respiratory motion (Figure 2, Panel c). A clearer comparison can be made by examining the 2D dose maps for the static and moving conditions without gating (Figure 2, Panels b and d). The gamma index dropped significantly to 32.6% (Figure 3, Panel b), with a maximum gamma index value of 8.58 and an average of 1.87. This large discrepancy highlights the impact of respiratory motion on dose distribution accuracy without gating.

In contrast, with the application of gating, the measured dose profile showed good agreement with the calculated values (Figure 2, Panel e). Additionally, the 2D dose map for the gated condition closely resembled that of the static condition (Figure 2, Panel f). The gamma index value remained high at 99.4%, with a maximum of 1.14 and an average of 0.32. The degradation in the gamma index observed in the non-gated moving condition was effectively prevented by applying gating (Figure 3, Panel c). All results related to the gamma index are summarized in Table 2.

**Table 2:** Gamma index analysis for static and motion conditions with and without gating. Results of the gamma index analysis (3 mm/2%) for the static, moving without gating, and moving with gating conditions.

Metrics	Static	Moving without gating	Moving with gating
Pass rate	99.4%	32.6%	99.4%
Average gamma index	0.3%	1.87%	0.32%
Maximum gamma index	1.23%	8.58%	1.14%

## Discussion

We developed the new motion platform named Itappachi and evaluated the dose distribution for the static condition, the moving condition without gating, and the moving condition with gating. Gating irradiation prevented dose degradation due to motion, and the dose was close to that for the static condition. Similar studies on VMAT dose measurement for both static and dynamic conditions have shown results consistent with ours [10-12]. This indicates that the specifications of the Itappachi platform are suitable for clinical applications.

As VMAT delivers a highly complex fluence map at each gantry angle, it is essential to evaluate 3D dose distributions rather than relying solely on 2D assessments. Dose measurement in a moving state is typically conducted during commissioning or for research purposes, rather than for routine patient-specific quality assurance. In scenarios such as excessive dose delivery to critical organs at risk or in the presence of an inhomogeneous density around the target, it is crucial to measure and compare the planned and actual dose distributions. However, it is problematic from a clinical standpoint that these dose measurements cannot be performed when the Delta4 Phantom+ or other dosimeters cannot be placed on commercially available motion platforms, hindering proper quality assurance management.

The upper and lower plates of the Itappachi platform are connected by embedded rails. This design element boasts multiple benefits. The rails minimize friction between parts for smoother operation, especially when dealing with heavy loads up to 56.8 kg, such as phantoms and dosimeters. The embedded rails of the platform significantly reduce the need for external lubricants compared to other commercially available platforms. The Itappachi platform is almost maintenance-free and simpler to operate. The reduced friction of the rails improves the accuracy of respiratory motion simulations. Improved precision in motion replication, crucial for dose measurements in dynamic conditions such as thoracoabdominal radiotherapy, is achieved through smoother movement. The lack of mechanical resistance of the platform contributes to its longevity and durability, making it a dependable tool for extended clinical applications. The impact of the rails on platform efficiency and clinical applicability is evident through their ability to deliver consistent, high-precision respiratory motion simulation outcomes. The design of the Itappachi platform allows for superior performance and addresses common challenges of other motion platforms, thereby enhancing quality assurance in modern radiotherapy.

In recent years, technological advancements in the medical field have rapidly progressed, with the introduction of the Internet of Things (IoT) being a significant breakthrough [13]. IoT plays a key role in thoracoabdominal radiotherapy by improving real-time data collection and enhancing treatment precision. IoT-enabled devices in the Internet of Medical Things allow for continuous monitoring of respiratory motion and patient positioning, as well as real-time dose adjustments, leading to greater accuracy in targeting moving areas. This integration also improves clinical workflows and quality assurance, while advanced analytics optimize treatment and improve patient outcomes, enhancing the effectiveness of cancer treatments.

## Conclusions

Our motion platform Itappachi offers a cost-effective and accurate solution for dose measurement under simulated respiratory motion. Its ease of use and high precision make it an essential tool for improving dose delivery accuracy in radiation therapy. We believe that the Itappachi platform shows great potential for improving quality assurance in dynamic treatment environments.
